# Treatment of Immune Thrombocytopenia: Contextualization from a Historical Perspective

**DOI:** 10.3390/hematolrep16030039

**Published:** 2024-06-26

**Authors:** Daniel Martínez-Carballeira, Ángel Bernardo, Alberto Caro, Inmaculada Soto, Laura Gutiérrez

**Affiliations:** 1 Department of Hematology, Hospital Universitario Central de Asturias (HUCA), 33011 Oviedo, Spain; angel.bernardo@sespa.es (Á.B.); alberto.caro@sespa.es (A.C.); inmaculada.soto@sespa.es (I.S.); 2 Platelet Research Lab, Instituto de Investigación Sanitaria del Principado de Asturias (ISPA), 33011 Oviedo, Spain; gutierrezglaura@uniovi.es; 3 Department of Medicine, University of Oviedo, 33006 Oviedo, Spain

**Keywords:** corticosteroids, fostamatinib, history, immune thrombocytopenia, ITP, IVIG, rituximab, splenectomy, thrombopoietin receptor agonist, treatment

## Abstract

Immune thrombocytopenia (ITP) is an autoimmune disease characterized by an isolated decrease in platelet count and an increased risk of bleeding. The pathogenesis is complex, affecting multiple components of the immune system and causing both peripheral destruction of platelets and inadequate production in the bone marrow. In this article, we review the treatment of ITP from a historical perspective, discussing first line and second line treatments, and management of refractory disease.

## 1. Introduction

Immune thrombocytopenia (ITP) is an autoimmune disease characterized by an isolated decrease in platelet count and an increased risk of bleeding. Primary ITP is defined as isolated thrombocytopenia < 100 × 10^9^/L in the absence of other causes. The term “secondary ITP” refers to all forms of immune thrombocytopenia except primary ITP [1]. Its annual incidence varies from 1.1 to 12.5/100,000 inhabitants/year [2]. Chronification is more common in adults, so the prevalence is higher in them (9.5–23.6 per 100,000 inhabitants) than in children (4.6 per 100,000 inhabitants) [3,4]. The predominant symptoms are skin and mucosal hemorrhages, although fatigue and predisposition to thrombosis are also part of its clinical spectrum [5].

The pathogenesis of the disease remains unknown, probably involving genetic and acquired factors. The pathophysiological mechanism is complex, affecting multiple components of the immune system and causing both peripheral destruction of platelets and inadequate production of them in the bone marrow [6,7]. The diagnosis of ITP continues to be one of exclusion, and the differential diagnosis of ITP must be made with the rest of the causes of thrombocytopenia, both secondary immune and non-immune [7]. We recently reviewed the pathophysiology, clinical manifestations, and diagnosis of ITP in another article of this issue [8]. In the current article, we focus on the treatment of ITP from a historical perspective, discussing first line and second line treatments, and management of refractory disease.

## 2. Werlhof’s Disease

Before platelets were discovered, the identification of ITP was based exclusively on the presence of purpura in an otherwise healthy individual. In 1735, a German physician and poet named Paul Gottlieb Werlhof provided the first detailed description of what we know today as ITP, separating it from the rest of the purpuras and naming it “morbus maculosus hemorrhagicus”. He described a case of a 16-year-old young woman with cutaneous hemorrhagic symptoms, epistaxis, and gingival bleeding that occurred after an infectious disease. At this time, the armamentarium of therapeutic options was obviously very limited. Phlebotomy was commonly prescribed for a number of conditions but was not considered appropriate in hemorrhagic disorders. Werlhof’s patient apparently recovered with Elixirium acidum halleri (citric acid), although it is more sensible to think of a spontaneous remission of the disease. Several decades later, Willan recommended “moderate exercise in the open air, a generous diet, and the free use of wine, …” for this disorder. Purgatives were also used extensively during the 19th century [9].

Throughout history, there was no scientifically based treatment for ITP until the beginning of the last century, with the advent of splenectomy. Since then, we have made great progress in our knowledge not only of the pathophysiology of the disease but also with respect to its management. Treatment has evolved from the surgical removal of a healthy organ to drugs currently in development that target specific molecular players of the different pathways involved in the pathogenesis of ITP (Table 1).

## 3. Current Treatment Modalities

**Figure 1:**
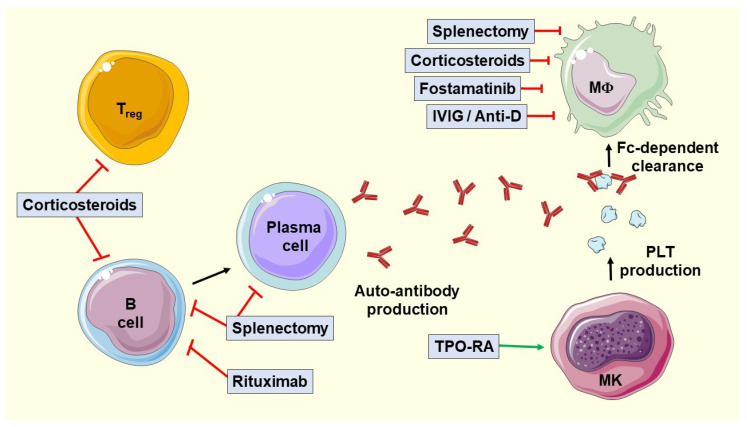
Current treatments for immune thrombocytopenia. The figure was generated using Servier Medical Art, provided by Servier and licensed under a Creative Commons Attribution 4.0 unported license (https://creativecommons.org/licenses/by/4.0/). Image adapted from: Singh A, Uzun G, Bakchoul T. Primary Immune Thrombocytopenia: Novel Insights into Pathophysiology and Disease Management. J Clin Med. 2021 feb 16;10(4):789 [5]. IVIG, intravenous immunoglobulin; MΦ, macrophage; MK, megakaryocyte; PLT, platelets; TPO-RA, thrombopoietin receptor agonist.

Although recommendations for the management of ITP are based on a low level of evidence, there are several guidelines and consensus documents that help physicians in the treatment of this entity. The first-line treatments for ITP continue to be corticosteroids and nonspecific intravenous immunoglobulins (IVIGs). Second-line therapy should be individualized, taking into account both comorbidities and the patient’s opinion. Thrombopoietin receptor agonists (TPO-RAs) have drastically changed the management of the disease, practically displacing splenectomy [11,12,13]. The different therapies currently in use are shown in Figure 1.

Treatment response criteria have not changed since they were standardized in 2009. The International Working Group (IWG) defines complete response (CR) as platelet counts ≥ 100 × 10^9^/L in the absence of bleeding; response (R) is defined as platelet counts ≥ 30 × 10^9^/L and <100 × 10^9^/L (with the condition that the platelet counts must be at least double the baseline platelet count) in the absence of bleeding; non-response (NR) is defined as platelet counts < 30 × 10^9^/L or less than twice the baseline value or the presence of bleeding [1].

## 4. Treatment Indications

The treatment of ITP has the following two main objectives: on the one hand, to maintain a platelet count that prevents severe bleeding, which is usually achieved with platelet counts greater than 30–50 × 10^9^/L, and, on the other hand, to avoid unnecessary treatments in asymptomatic patients, since the side effects may be more important than the complications derived from the disease itself. Therefore, treatment should be individualized, and the goal should not be to normalize the platelet count. When choosing treatment, the least toxic and the lowest possible dose should be used [13].

In a systematic review that included 118 studies and a total of 10,908 patients, the risk of intracranial hemorrhage was found to be 1.0% overall (1.4% in adults and 0.4% in children) [14]. The risk of death from bleeding in adults with severe chronic ITP was estimated in an analysis that included 1817 patients from several published studies. Annual mortality from bleeding was 1.6–3.9 cases per 100 patient years, with the risks of fatal bleeding and severe non-fatal bleeding increasing with age [15].

The decision to start treatment in newly diagnosed ITP depends on several factors, with the platelet count being the main acknowledged parameter to assess the bleeding risk. Compared to chronic ITP patients with normal platelet counts, those with a count between 25 and 49 × 10^9^/L and <25 × 10^9^/L have 2.5-fold and 7-fold higher one-year risks of bleeding requiring hospital contact, respectively [16]. In addition to the platelet count, other patient-related factors may help to determine the bleeding risk, such as age (>65 years), prior bleeding events, comorbidities associated with high bleeding risk (e.g., hypertension or cerebrovascular disease), kidney or liver failure, anticoagulant and antiplatelet medication, surgical interventions, and lifestyle [5,16,17].

Current guidelines recommend starting treatment below 20–30 × 10^9^/L platelets, regardless of the presence of bleeding. If the platelet count is between 20–30 × 10^9^/L and 50 × 10^9^/L, it is not recommended to initiate treatment in the absence of bleeding, although it may be necessary in special circumstances, such as in the case of hemorrhagic symptoms, surgery, or the need for antiplatelet treatment or anticoagulation. With more than 50 × 10^9^/L platelets, treatment is generally not indicated, and in the case of bleeding, it is recommended to look for other causes that justify it. Above this threshold, treatment may be considered only if the patient requires intracranial or ocular surgery or has platelet dysfunction [11,12,13].

When it comes to the decision to hospitalize, multiple variables must be considered, such as the stage of the disease, platelet counts, and the patient’s opinion. The scientific evidence to establish hospitalization criteria is scarce and, above all, based on the risk of potentially severe hemorrhagic complications. Hospital admission is recommended for adults with recently diagnosed ITP and a platelet count less than 20 × 10^9^/L, even if they are asymptomatic or with minimal mucocutaneous bleeding. If these circumstances occur in patients with already established ITP, management can be outpatient. Regardless of the stage of the disease and platelet count, patients with severe bleeding should be hospitalized [11,12,13].

## 5. First-Line Treatment

### 5.1. Glucocorticoids

The increase in platelet counts with glucocorticoids, adrenocorticotropic hormone (ACTH), or other immunosuppressive agents has been described since 1951 [18]. The time required for response and the side effects associated with prolonged treatment prevented these agents from becoming first-line medication. The first randomized double-blind study that compared corticosteroids with placebo for 21 days was carried out in children with recently diagnosed ITP by Swiss Joerg Sartorius in 1972. The results showed a more rapid increase in platelet count in children treated with prednisolone compared to those given placebo. However, forty days later, significant differences between the two groups were no longer evident. Prednisolone became the first-line treatment for newly diagnosed ITP [10]. The use of dexamethasone (DXM) in the management of ITP was first reported in 1994 by Judith Andersen, who described 10 adult patients with refractory ITP who responded to six cycles of DXM 40 mg/day for 4 days [19]. Since then, several prospective, multicenter, and randomized studies have compared prednisone (PRD) and DXM, the most used glucocorticoids today, with good results.

Glucocorticoids bind to cytosolic receptors and modulate a wide variety of genes, triggering numerous physiological changes. Their immunosuppressive action results from the modulation of B-cell activation through a decrease in B-cell activating factor (BAFF) and the modulation of dendritic cells. In ITP, their main effect is to decrease platelet clearance by inhibiting the expression of Fc receptors in immune cells [20].

The most common dose of PRD is 1 mg/kg/day. It is of great importance to limit the duration of treatment in order to reduce side effects. In this sense, the maximum time with the initial dose of prednisone is 3 weeks, after which the dose must be reduced progressively to be withdrawn 6 weeks from the start (8 weeks maximum). Occasionally, some patients may benefit from prolonged low-dose corticosteroids (prednisone ≤ 5 mg/day). The most used regimen of DXM is 40 mg/day for 4 days every 2 weeks, up to a maximum of 3 cycles; it is a better defined treatment and shorter in time [11,12,13].

In a meta-analysis of several randomized studies that compared DXM (3 cycles of 40 mg/day × 4 days) and PRD (1 mg/kg/day 14–28 days with subsequent progressive decrease), after 14 days of treatment, the rate of overall responses was slightly higher in the DXM group (79% vs. 59%, RR 1.22, 95% CI 1.00–1.49, *p* = 0.048). However, no differences were observed in the overall response at 6 months (54% vs. 43%, RR 1.16, 95% CI 0.79–1.71, *p* = 0.44). Therefore, DXM seems to be associated with faster responses than PRD but a similar sustained response [21]. It is worth noting that DXM may have a better safety profile (lower rates of Cushing’s disease, weight gain, and infections) compared to prednisone [22].

In general, the response to corticosteroids is observed within the first 2 weeks of treatment [1]. Although 60–80% of ITP patients have an initial response to glucocorticoids, only 30–50% of adults have a sustained response after discontinuation [23]. As a predictor of response, Wang et al. found that the presence of anti-GPIb/IX antibodies predicted a poor initial response to corticosteroids, although there was no correlation with a sustained response later [24]. More studies are needed to determine the role of antiplatelet antibodies in this regard. 

### 5.2. Intravenous Immunoglobulins (IVIGs)

Until 1980, the classic management of ITP consisted mainly of treating bleeding episodes with corticosteroids. In patients with refractory disease, splenectomy or immunosuppressive or cytostatic treatments were indicated on an individualized basis [25]. The introduction of IVIGs by Imbach et al. in 1981 was a milestone in the treatment of symptomatic ITP in children [26]. Later, in 1983, their effectiveness was validated in adults [27] and, in 1984, in pregnant women [28].

IVIGs are purified from the plasma of healthy donors and mainly contain polyclonal IgG [25]. Several mechanisms of action of IVIGs have been postulated in ITP, including blockade/inhibition of the mononuclear phagocytic system, neutralization of autoantibodies by anti-idiotype antibodies, elimination of pathogenic autoantibodies due to competitive inhibition of the neonatal Fc receptor, cytokine modulation, complement neutralization, and immune complex formation leading to dendritic cell priming [29].

IVIGs (whether or not associated with corticosteroids) are used in patients with severe bleeding or the need to rapidly increase the platelet count. IVIGs increase the platelet count in 1–4 days in 80% of patients, but their effect is temporary and lasts only 1–2 weeks [30]. Concomitant use of glucocorticoids with IVIGs may be associated with a more sustained response compared to IVIGs alone [31]. Therefore, ideally, corticosteroids should always be associated with IVIGs, unless they are contraindicated. In some patients with contraindications to high doses of corticosteroids (poorly controlled diabetes, psychiatric disorders. or active infection), IVIGs without corticosteroids should be the first line of treatment. The doses used are 1 g/kg/day × 2 days or 0.4 g/kg/day × 5 days [13]. Adverse effects occur in about 5% of individuals and can be reduced by decreasing the infusion rate. Symptoms are mostly mild and consist of chills, headache, myalgia, nausea, and fever. Acute aseptic meningitis after administration is rare and transient. Thrombotic events are associated with high solution osmolality and recipient serum viscosity [25]. A potentially fatal complication is anaphylaxis in patients with severe IgA deficiency. Routine dosing of immunoglobulins upon diagnosis of ITP would allow these patients to be identified and, thus, reduce the risk.

The antigenic target of platelet autoantibodies may influence the response profile of IVIGs, as suggested by a small study with 17 adult patients with ITP where anti-GPIb/IX antibodies were associated with a worse response to IVIGs [32]. In a retrospective study carried out years later with 156 adult patients with untreated ITP, treatment with IVIGs showed a response (platelet count ≥ 30 × 10^9^/L) in only 36.4% of patients with anti-GPIb/IX autoantibodies, while the response in those who were negative for anti-GPIb/IX was 80%. The presence of anti-IIb/IIIa did not influence the efficacy of IVIGs [33]. Experiments with animals have shown that the destruction of platelets in this case could be related to a phenomenon of desialylation and premature senescence that leads to their destruction through the hepatic Ashwell–Morell receptor and not to platelet destruction through phagocytosis mediated by the Fc receptor expressed on the surface of splenic macrophages [34]. However, these data have been challenged recently, since other researchers failed to show the relationship between the specificity of autoantibodies and the lack of response to IVIGs [35]. 

### 5.3. Anti-D Immunoglobulin

In 1983, A. Salama and collaborators were the first to document a platelet response in Rh-positive patients with ITP after the intravenous administration of specific anti-D immunoglobulin; they also introduced the concept of macrophage blockade [36,37]. In the following years, more publications about this therapy emerged [38,39,40,41,42,43].

Anti-D immunoglobulin consists of IgG selectively derived from plasma of donors immunized against the D antigen [44]. It selectively binds to Rh-positive red blood cells, and these sensitized red blood cells competitively inhibit platelet destruction by occupying Fc receptors on splenic phagocytes [38]. Therefore, specific anti-D IgG is only effective in non-splenectomized Rh-positive patients. International consensus guidelines approve the use of IV anti-D immunoglobulin in first-line treatment for ITP [13]. The overall response rate is 65%, and the median response time is 3 days [45]. A single intravenous dose of 50–75 µg/kg is recommended [46]. Side effects include mild infusion reactions such as headache, nausea, chills, and fever [43]. However, life-threatening episodes of severe intravascular hemolysis and disseminated intravascular coagulation have also been reported [47,48]. For safety reasons, anti-D was withdrawn from European markets in 2009.

## 6. Second-Line Treatment

The four currently used second-line treatment modalities are compared in Table 2. We discuss them in detail below.

### 6.1. Thrombopoietin Receptor Agonists

Thrombopoietin (TPO) is the most potent regulatory molecule of megakaryopoiesis. It is a protein of 353 amino acids that binds through its amino-terminal domain to its receptor MPL (“myeloproliferative leukemia protein”), so named because it was originally identified as the homolog proto-oncogene to the oncogene of the murine myeloproliferative leukemia virus *c-Mpl*. The *MPL* gene was cloned in 1992 at a time when the corresponding “MPL ligand” was unknown [49]. It was in 1994, two years later, when TPO was cloned [50,51,52]. MPL is present in a wide variety of hematopoietic tissues ranging from stem cells to megakaryocyte colony-forming cells (Meg-CFC), myeloid and erythroid progenitors, early and late megakaryocytes, and mature platelets [53].

In 1996, it had already been observed that TPO levels in ITP were decreased compared to central thrombocytopenias such as bone marrow aplasia [54,55]. This finding suggested a potential defect in the stimulation of megakaryopoiesis that, together with the recent characterization of TPO, gave rise to a new therapeutic approach in ITP consisting of the stimulation of platelet production. This began the clinical development of first-generation TPO-RAs comprising two recombinant thrombopoietins, namely recombinant human thrombopoietin (rhTPO) and pegylated, recombinant human megakaryocyte growth and development factor (PEG-rhMGDF). The two of them are potent stimulators of megakaryocyte growth in both animal models and humans, and several clinical trials have demonstrated improvement in platelet counts in a variety of clinical situations ranging from chemotherapy-induced thrombocytopenia to ITP. Unfortunately, some healthy volunteers developed antibodies against PEG-rhMGDF, neutralizing its activity and cross-reacting with neutralizing endogenous thrombopoietin, producing paradoxical thrombocytopenia. This concern about their antigenicity led to the discontinuation of any further development of recombinant thrombopoietins [53].

Given the promising results with these two molecules, second-generation TPO-RAs subsequently emerged, which were specifically developed to have no homology with endogenous TPO. In 2008, the era of agonists began with the approval of eltrombopag and romiplostim for the treatment of ITP in the USA. Since then, its use has expanded throughout the world, bringing about a revolution in the management of patients in whom corticosteroids fail. 

Both romiplostim and eltrombopag bind to the TPO receptor, causing a conformational change in the receptor; activation of the JAK2/STAT pathway; and, as a result, increased proliferation of megakaryocyte progenitors and increased production of platelets. It has also been described that both TPO-RAs have immunomodulatory capacity, increasing the regulatory effects of T and B cells. It has been suggested that this effect is mediated by TGF-B, a cytokine relevant to the development of regulatory T cells, and that it is found in abundance in megakaryocytes and platelets. It is not yet known whether these potential immunomodulatory effects result in the durable treatment-free responses being seen with both TPO-RAs [56].

In randomized, placebo-controlled clinical trials in patients with chronic ITP who failed at least one prior therapy, 70 to 95% of patients had an increase in platelet count after initiation of treatment with these agents, and 40–60% had lasting responses. Although these two agents have not been compared directly, response rates appear to be similar [23]. Both agonists are indicated in ITP patients ≥ 1 year of age who do not respond to other treatments (e.g., corticosteroids or immunoglobulins). Additionally, eltrombopag is indicated in adult patients with chronic hepatitis C virus infection for the treatment of thrombocytopenia, where the degree of thrombocytopenia is the main factor preventing the initiation or limiting the ability to maintain optimal interferon-based therapy. Eltrombopag is also indicated in adult patients with acquired severe aplastic anemia who were either refractory to prior immunosuppressive therapy or heavily pretreated and unsuitable for hematopoietic stem cell transplantation [57,58].

Romiplostim is a recombinant fusion protein formed by the Fc fraction of human IgG_1_ (IgG_1_) linked to a TPO receptor binding domain. Romiplostim and endogenous TPO have the same TPO receptor binding site, i.e., the extracellular domain, suggesting a common mechanism of action [59]. Romiplostim is administered weekly subcutaneously, with the recommended initial dose being 1 μg/Kg weekly with increments of 1 μg/Kg depending on the platelet count to maintain a platelet count ≥ 50 × 10^9^/L. The maximum recommended dose is 10 μg/Kg/week [57]. The great drawback of the drug is its parenteral administration.

The efficacy and safety of romiplostim was observed in two parallel phase III studies carried out in Europe and the USA that recruited 125 patients with severe refractory ITP. The overall platelet response rate (either durable or transient platelet response) was observed in 88% of non-splenectomized patients and 79% of splenectomized patients treated with romiplostim, compared with 14% of non-splenectomized patients and 0% of splenectomized patients who received placebo [60]. The results of a long-term study that included 292 adult patients (splenectomized or not) with a follow-up of up to 5 years showed a response (≥50 × 10^9^/L platelets at least once) in 95% of patients, with a response maintained by all of them in a median of 92% of study visits [61]. When the data of the 1111 ITP patients treated with romiplostim included in the 13 studies (phase II/III and extension) were analyzed, the response rate (≥50 × 10^9^/L platelets at least once) was 82% and 91% in splenectomized and non-splenectomized patients, respectively. The observed sustained response (≥50 × 10^9^/L platelets for 9 of 12 weeks) was 68% in splenectomized patients and 80% in non-splenectomized patients [62]. 

Eltrombopag is a small non-peptide molecule that binds to TPO-R and activates the JAK2/STAT signaling cascade, increasing platelet production. Unlike endogenous TPO, eltrombopag binds to the transmembrane domain of TPO-R and, therefore, does not compete with it to bind to the receptor and may enhance the function of endogenous TPO instead of replacing it, as suggested by in vitro studies [59]. It is administered orally in the form of tablets, and the recommended starting dose is 50 mg per day, which can be increased to a maximum of 75 mg per day, with the objective of maintaining ≥50 × 10^9^/L platelets. In patients of Asian origin or with liver failure, the recommended initial dose is 25 mg per day. The tablets should be taken at least two hours before or four hours after any of the following products, as it has been documented that eltrombopag has chelating activity: antacids, dairy products (or other foods containing calcium), or mineral supplements containing polyvalent cations (e.g., iron, calcium, magnesium, aluminum, selenium, and zinc) [58].

In the first prospective, double-blind, randomized phase III study, eltrombopag was compared with placebo for 6 weeks in patients with chronic ITP. The results demonstrated a response rate of 59% in the eltrombopag arm versus 16% in the placebo arm [63]. Subsequently, the RAISE study was developed, also a randomized and double-blind phase III trial, where eltrombopag was again compared with placebo in 197 patients with chronic ITP, in this case with a follow-up period of 6 months. A proportion of 79% of patients achieved response in the agonist arm compared to 28% in the placebo group [64]. The EXTEND study included 302 patients to evaluate the long-term efficacy and safety of the drug (time period of up to 8.8 years). It concluded that 85.8% of the patients achieved a response, this percentage being lower in splenectomized patients than in non-splenectomized patients [65].

In the pediatric population, four studies have been carried out with romiplostim [66,67,68,69] and two with eltrombopag [70,71], showing similar efficacy and safety profiles to adults. 

#### 6.1.1. Adverse Events

The most frequently observed adverse effects of romiplostim are headache and fatigue [61]. The most common side effects of eltrombopag are headache, gastrointestinal symptoms (nausea, vomiting, and diarrhea), and mild elevations in transaminases [64].

The most worrying adverse events are thromboembolism and bone marrow fibrosis, which are considered a class effect, so they can occur with both agonists. In extension studies of both drugs, thromboembolism (arterial or venous) developed in 6% of patients during a median follow-up of 2 years. The events occurred predominantly in patients with other risk factors [61,65]. Although not corroborated in adequately designed trials, annualized thrombosis rates in adults appear to be 2–3 times higher with TPO-RA than in the ITP population not treated with TPO-RA and even higher when compared to the control population without ITP. On the other hand, most of the available data on the risk of thrombosis are based on retrospective studies and registries, which probably underestimate the risk of thrombosis in the ITP population. Thrombotic events are not associated with thrombocytosis or a higher dose of the agonist, and at least 30–50% of cases occur in patients with platelet counts below the normal threshold. In general, thrombosis tends to occur in the first year of treatment. The pathogenic mechanisms responsible for the increased thrombotic risk related to TPO-RA have not yet been identified. As a precaution, the patient’s individual risk profile should be considered when initiating treatment with a TPO-RA to assess whether the expected reduction in bleeding risk outweighs the risk of thrombosis [56].

In the first studies carried out with agonists, concerns arose about the possible induction of bone marrow fibrosis due to sustained stimulation of megakaryopoiesis; however, these effects have not been confirmed in more than 10 years of clinical experience. Most cases of fibrosis observed in patients with ITP receiving TPO-RA are mild, with moderate reticulin fibrosis observed in less than 10% of patients, which is reversible upon discontinuation. Severe fibrosis was extremely rare in all studies. Fibrosis was not associated with the type, dose, or duration of treatment. In general, agonists do not appear to induce substantial fibrosis or changes in blood cell number or morphology. Currently, there is no consensus on whether or how to monitor bone marrow fibrosis in patients with ITP receiving TPO-RA. Almost no center performs routine marrow biopsies in patients treated with TPO-RA; however, if a biopsy is performed and severe reticulin or collagen fibrosis (MF-3) is discovered, it is recommended to discontinue TPO-RA. With moderate fibrosis (MF-2), the patient can continue with TPO-RA, but an additional biopsy may be necessary after six months [56]. Our clinical practice is to perform bone marrow biopsy in those patients under treatment with agonists who, during follow-up, present suspicious data such as associated cytopenias, an unexplained increase in LDH, splenomegaly, or morphological abnormalities in peripheral blood (for example, dacrocytes).

Another relevant adverse event, in this case associated only with eltrombopag, is hepatotoxicity. In a drug extension study, patients developed hepatobiliary alterations (increased transaminases or bilirubin) in 15% of cases (none grade 4), with a median duration of treatment with eltrombopag greater than 2 years [65]. Bilirubin elevations primarily involve unconjugated bilirubin (which is not indicative of severe liver damage). These alterations are mostly asymptomatic and reversible with drug interruption or dose reduction. Liver impairment usually occurs during the first year of treatment, warranting regular monitoring of liver enzymes at more frequent intervals during the first years of treatment [56].

Lastly, given that the TPO receptor is expressed in hematopoietic stem cells, there is always concern about the possibility of triggering a neoplasia or promoting an existing associated one. A large analysis of pooled data from 13 clinical trials of ITP patients treated with romiplostim showed that rates of hematological and non-hematological malignancies were similar between the agonist group and the placebo or “standard-of-care” group (any treatment for ITP other than romiplostim) [72].

#### 6.1.2. Agonist Change (Switch)

Given that the two TPO-RAs have similar efficacy and safety, the choice of one or the other depends on the patient’s preferences agreed upon with the doctor, mainly assessing the route of administration or the frequency of necessary visits to the hospital. 

Limited data from observational studies suggest that if one agonist is switched for another, it produces a platelet response in 50–80% of patients [73,74,75], with a lower response when the reason for the change is lack of efficacy compared to a different reason (platelet fluctuation, patient preference, or adverse effects) [76]. In a study carried out in our country, in which the results of the use of TPO-RA in clinical practice were evaluated, it was observed that 38.8% of patients initially treated with eltrombopag switched to romiplostim, and 24.1% of those receiving romiplostim switched to eltrombopag. The main reason for the change was the lack of efficacy in the case of those initially treated with eltrombopag and the preference of the patient or doctor in the case of those treated with romiplostim [77].

#### 6.1.3. TPO-RA Discontinuation

Once we have achieved a response with an agonist, continuing treatment is usually required to maintain it. However, in retrospective and prospective cohort studies, it has been observed that 10 to 30% of patients can discontinue treatment, keeping the disease in remission [78,79]. This situation is called sustained remission off-treatment (SROT) which may be due to immunomodulatory actions performed by this therapeutic group. This observation encouraged some practitioners to progressively reduce the TPO-RA dose and, ultimately, to suspend treatment, provided that a drop in the platelet count was not detected. Normally, candidates for achieving SROT after progressive dose reduction followed by discontinuation are those who present with stable platelet counts (50–100 × 10^9^/L) during a 4–6-month period on TPO-RA treatment, regardless of disease stage [11].

#### 6.1.4. Avatrombopag

Avatrombopag is a new TPO-RA that binds to the transmembrane region of the TPO receptor in a similar way to eltrombopag. Unlike this, avatrombopag can be administered without dietary restrictions and does not require monitoring of liver function. A phase III clinical trial in adults with chronic ITP showed more median weeks of response during the first 26 weeks in patients receiving avatrombopag compared to those receiving placebo (12.4 vs. 0.0 weeks, respectively). The response rate at day 8 was 65% with avatrombopag compared with 0% in the placebo group. The most common side effects were headache, arthralgia, and fatigue [80]. It is probably associated with an increased thrombotic risk as a class adverse effect, since in the four pooled clinical trials carried out in chronic ITP, thromboembolic events were observed in 7% (9/128) of patients [81], a rate similar to that reported in long-term studies of romiplostim and eltrombopag. More long-term and real-life evidence is needed for a better evaluation of the safety of avatrombopag. 

Based on the good obtained results, avatrombopag was approved by the FDA in 2019 and by the EMA in 2020 for the treatment of chronic primary ITP in adult patients who are refractory to other treatments (e.g., corticosteroids or immunoglobulins). The recommended starting dose is 20 mg (one tablet) once a day with food. The platelet count usually increases within 1 week after the start of treatment and decreases within 1 to 2 weeks after its discontinuation. It is recommended to use the lowest dose necessary to maintain a platelet count ≥ 50 × 10^9^/L. The maximum recommended daily dose is 40 mg (two tablets). Avatrombopag should be discontinued if the platelet count does not increase to ≥50 × 10^9^/L after 4 weeks of treatment at the maximum daily dose [81].

A retrospective observational study of adults with ITP who switched from eltrombopag or romiplostim to avatrombopag showed a platelet response in 41/44 patients (93%). The platelet response was also achieved in 12 (86%) of the 14 patients in whom the reason for the change had been the ineffectiveness of the previous agonist [82]. These results make it a very good option to make the switch. 

### 6.2. Fostamatinib

Fostamatinib is an oral inhibitor of splenic tyrosine kinase (SYK) involved in the FcR signaling pathway. Thus, it reduces platelet clearance through splenic Fc receptors. Pooled analyses of two-phase III clinical trials in patients with persistent/chronic refractory ITP (median disease duration of 8.5 years) showed an overall response (≥1 platelet count ≥ 50 × 10^9^/L within the first 12 weeks) in 43% of patients treated with fostamatinib compared to 14% of those receiving placebo. The median time to response was 15 days. A stable response (platelets ≥ 50 × 10^9^/L at ≥4 of 6 biweekly visits, weeks 14–24) was seen in 18% of patients in the fostamatinib group compared to 2% of those receiving placebo [83]. In a post hoc analysis of a phase III and extension study, a higher platelet response rate (78% vs. 48%) and fewer bleeding events (28% vs. 45%) were observed when fostamatinib was used as second-line treatment compared to its use as a third-line or later treatment [84]. 

Fostamatinib was approved by the FDA in 2018 and the EMA in 2020 for the treatment of chronic ITP in adults who are refractory to other treatments. The recommended starting dose is 100 mg twice daily and may be increased to 150 mg twice daily if the platelet count has not increased to at least 50 × 10^9^/L after 4 weeks. Treatment should be discontinued after 12 weeks if the platelet count does not increase to a level sufficient to prevent clinically significant bleeding [85]. The most common adverse reactions are diarrhea (31%), hypertension (28%), and nausea (19%) [83].

SYK is also expressed in platelets, with ligation of three receptors, namely GPVI, CLEC2, and FcγRIIA, leading to SYK-mediated signaling. All three receptors are involved in thrombosis, while playing negligible roles in hemostasis, and fostamatinib targets these three platelet receptors without affecting others involved in hemostasis, such as protease-activated receptor-1 (PAR1) for thrombin and ADP receptor P2Y12. In in vitro experiments, R406, an active metabolite of fostamatinib, inhibited FcγRIIA-mediated platelet aggregation induced by heparin-induced thrombocytopenia (HIT) sera, GPVI-mediated platelet aggregation induced by collagen, and CLEC2-mediated platelet aggregation induced by podoplanin but had no effect on ADP-mediated platelet aggregation. Thus, R406 inhibited signaling via the receptors that are more predominantly involved in thrombosis, leaving hemostasis-specific signaling unaffected. In vivo use of R406 did not result in prolongation of bleeding time in mice. Testing fostamatinib in healthy volunteers at doses that are much higher than those typically prescribed to ITP patients did not affect platelet aggregation ex vivo. Thus, evidence indicates that fostamatinib may reduce the risk of thrombosis without affecting hemostasis or increasing the bleeding risk [86]. However, the fact that, as mentioned above, fewer bleeding events were observed when fostamatinib was used as second-line treatment compared to its use as a third-line or later treatment suggests a potential differential susceptibility of platelets towards the action of this inhibitor. In a previous study conducted in our lab, we characterized the platelet proteomes of different murine models of ITP; we observed downregulation of SYK in platelets from the passive ITP model (which reflects acute ITP) and partial restoration of SYK levels in platelets from the active ITP model (which reflects chronic ITP). Extrapolated to humans, our results may explain why fostamatinib is safer in terms of bleeding as a second-line treatment. It does not affect platelet functionality through SYK inhibition because it is already downregulated in newly diagnosed ITP. However, variation in SYK levels in chronic ITP may result in an increased risk of bleeding events and reduced clinical responses [87]. Certainly, such results should be confirmed with human samples.

Fostamatinib is currently the second-line treatment of choice when agonists are not appropriate due to high thrombotic risk (e.g., patients with COVID-19) and is also preferred over rituximab or other immunosuppressants in patients with high infectious risk [88]. 

### 6.3. Rituximab

Rituximab (RTX) is a genetically engineered chimeric mouse/human monoclonal antibody representing a glycosylated immunoglobulin with human IgG_1_ constant regions and murine light-chain and heavy-chain variable region sequences [89]. It is directed against CD20, a membrane glycoprotein expressed on the surface of mature B cells. It induces apoptosis of B lymphocytes and destruction in the spleen through cytotoxicity mediated by the complement or antibody-dependent pathway. The result is a rapid (in the first week) and profound depletion of B lymphocytes that translates into a decrease in antibody titers. The B-lymphocyte count in peripheral blood remains low for at least 6–12 months after administration. It seems that the action of RTX is not only limited to humoral immunity but can induce indirect regulation of the T-cell compartment [20,90].

It was introduced for the treatment of B lymphomas in the late 1980s [91]. The first case in which RTX was used for the treatment of an autoimmune disease was published in 1998; it involved a patient with cold agglutinin disease and an IgM paraprotein associated with non-Hodgkin lymphoma who was successfully treated with four weekly infusions of rituximab [92]. In 2001, the first successful case of RTX in the treatment of ITP associated with low-grade non-Hodgkin lymphoma was published [93]. Subsequently, it has also begun to be widely used for the treatment of autoimmune manifestations associated with lymphoproliferative disorders and, as it is a therapy borrowed from lymphomas, a dosage regimen of 375 mg/m^2^ weekly for 4 weeks became the “standard dose” in autoimmune diseases [90]. Since then, RTX began to be used in multiple autoimmune diseases, such as systemic lupus erythematosus, rheumatoid arthritis, and autoimmune hemolytic anemia, among others. Despite multiple studies and extensive experience, the only autoimmune diseases for which it is currently approved by the FDA and EMA and, therefore, indicated according to the technical specifications are rheumatoid arthritis, granulomatosis with polyangiitis, microscopic polyangiitis, and pemphigus vulgaris [89]. 

In 2001, Stasi et al. published the first prospective study of RTX use in ITP in which 25 adult patients with chronic ITP were treated with four weekly infusions of RTX at a dose of 375 mg/m^2^. The overall response rate was 52%, with 28% sustained responses [94]. The use of RTX expanded to pediatric ITP. The first large series with children was published in 2005 and included 24 patients with refractory chronic ITP or relapse after previous treatments. The overall response to standard-dose RTX was 79%, and the sustained response rate was 37% [95]. Multiple studies were subsequently carried out, both in children and adults. In adults, the overall response rate is 57% and reduces to 38% in the first year and 21% in the fifth year. In children, effectiveness is similar to that in adults, with initial overall response rates of 57% and sustained 5-year responses of 26% [96].

The standard dose produces a marked reduction in malignant and non-malignant B cells in peripheral blood and bone marrow. Since the total B-cell mass is much smaller in ITP patients than in lymphoma patients, it was unclear whether a lower dose could be equally effective [90]. Schemes with doses of 100 mg weekly for 4 weeks have been described, which seem to be just as effective as the standard dose [97,98]. On the other hand, a French prospective registry of adult patients with ITP who received RTX in real life compared “standard doses” with the approved dose for rheumatoid arthritis (two infusions of 1 g separated by 2 weeks). No differences were observed between the two regimens in either initial or long-term response [99]. Another French study compared 61 adult ITP patients receiving standard-dose RTX with 46 patients receiving the rheumatoid arthritis dose, concluding that high-dose RTX is effective and safe in the treatment of ITP [100]. Therefore, both low and high doses of RTX appear to be just as effective as the standard dose.

Two studies also evaluated the early use of RTX as a first-line treatment in addition to corticosteroids. In both, the administration of DXM (40 mg/day × 4 days) in monotherapy was compared with the administration of DXM together with the standard dose of RTX in adult patients. In the study by Zaja et al. sustained 6-month response rates of 63% for the combination treatment and 36% for the dexamethasone monotherapy group were observed [101], compared to 58% and 37%, respectively, in the study by Gudbrandsdottir [102].

There are no clear predictors of response to RTX, although some studies found that young women with a disease duration of less than 2 years had a greater and longer-lasting response [103]. The role of platelet autoantibodies in the response to RTX is controversial [90].

The most notable adverse events associated with RTX are those related to the first infusion (nausea, chills, hypertension, rash, fever, pruritus, throat irritation, and hypotension); they are generally mild and transient, becoming more infrequent with subsequent doses and reduced and limited with premedication. In a French prospective study, the most frequent adverse events were those related to the infusion (in 15% of patients), and the majority were mild [99].

One of the main long-term concerns is the risk of infection, which is rare. In a study by Khellaf et al., infections were observed in seven patients (3%), the most serious being in patients over 70 years of age with comorbidities [99]. A meta-analysis that included five clinical trials and 463 ITP patients treated with RTX found no increased risk of infection [104]. Reactivation of hepatitis B has been described in asymptomatic carriers of the virus, so it is an aspect that must be considered before deciding to start treatment with RTX. A rare but potentially fatal infection that has been linked to rituximab is progressive multifocal leukoencephalopathy (PML) caused by the activation of John Cunningham (JC) virus and its spread to the central nervous system. This complication has been reported almost only in patients with lymphoproliferative disorders, and only very few cases have been observed in patients with autoimmune diseases, especially systemic lupus erythematosus. Furthermore, patients who develop PML are often profoundly immunocompromised by combination chemotherapy, and rituximab is usually only one of many drugs they receive. There is only one well-studied case of PML in ITP that occurred more than 3 years after RTX exposure [90].

### 6.4. Splenectomy

Splenectomy was the first scientifically based treatment for ITP. In 1916, a Viennese medical student named Paul Kaznelson reported the first case undergoing splenectomy with a good response, a woman with a history consistent with chronic ITP [105]. In subsequent years, several case series were reported, beginning the era of splenectomy. It was the only treatment available for ITP until the 1950s, when the era of immunosuppressants began after the famous Harrington experiment. Harrington transfused himself with blood from an ITP patient. Harrington’s platelet count, previously normal, dropped sharply and returned to normal over the following days. The same experiment was subsequently carried out with healthy volunteers, with the same result. He concluded that the platelet clearance in ITP was due to a “humoral factor” [106]. Even so, splenectomy continued to be the reference therapy for chronic ITP and second-line standard until the appearance of TPO-RA at the beginning of the 21st century, which gradually displaced it almost completely during the second decade of this century [107].

Splenectomy is traditionally considered the treatment that offers the highest rate of sustained responses, since the spleen is the main site of autoantibody production and platelet destruction. The overall response rate is greater than 80% and 50% to 75% maintained at 5 years. Post-splenectomy relapses occur in 20–30% of patients, often within the first 24 months [108]. There are no clear predictors of response [109], although knowing the main site of platelet clearance, either by platelet kinetics or by the degree of sialylation, can help predict the success of splenectomy.

The main complications associated with splenectomy are postoperative hemorrhage, infection by encapsulated bacteria, sepsis, and venous or arterial thrombosis [108]. Compared with open surgery, laparoscopic surgery has lower postoperative morbidity and mortality rates. In a systematic review of more than 3000 splenectomies for ITP, the laparoscopic approach had lower mortality (0.2% vs. 1.0%) and a lower complication rate (9.6% vs. 12.9%) than open surgery [109]. To reduce the risk of venous thrombosis, prophylactic anticoagulation with low-molecular-weight heparin is usually recommended after surgery according to the patient’s characteristics, always after assessing the patient’s platelet count and other bleeding risk factors [11]. A large study showed an increased risk of early and late sepsis among ITP patients who had undergone a splenectomy compared to those who had not been splenectomized [110]. Of special interest is the risk of infection by encapsulated bacteria, especially in the first months after surgery, which is why vaccination is recommended against Haemophilus influenzae type B, Neisseria meningitidis (meningococcus), and Streptococcus pneumoniae (pneumococcus) at least 2 weeks before, with pertinent revaccinations [11,13].

Splenectomy is generally not performed on older patients due to an increased risk of relapse and complications [109,111]. Currently, splenectomy continues to play a role in adult patients with ITP refractory to several lines of medical treatment. In a recently published French study, among adults patients with chronic ITP who had previously failed both TPO-RAs and rituximab treatment, the sustained response rate after splenectomy was 46% [112]. Thus, success rates are slightly lower, as expected, than those historically reported with data from the pre-agonist era. It is recommended to wait at least 12–24 months from diagnosis before performing splenectomy, given the possibility of remission [11,13]. In children, it is indicated on very rare occasions and only when all available medical therapies are exhausted. The basis for such recommendations is the natural history of the disease itself. Although more than half of adults relapse or do not respond to corticosteroids and the trend is towards chronification, 16.2% of patients with persistent ITP will still normalize their platelet count without treatment at 12 months [113]. As for children, in the majority of patients, the clinical course is acute and, furthermore, among those whose ITP persists at 6 months, 25% will recover their platelet count at 12 months; recoveries have been described even beyond the year of diagnosis [114].

## 7. Refractory ITP

In 2009, the international consensus group initially defined refractory ITP as occurring when the patient (i) does not respond or relapses after splenectomy and (ii) has severe ITP or has a risk of bleeding that requires therapy [1]. Since splenectomy is used less and less today, this definition is outdated. In 2020, Miltiadous et al. defined refractory ITP as the lack of response to ≥2 treatments, with no single drug to which it responds and with a very low platelet count accompanied by bleeding. In this way, the need for splenectomy to consider ITP as refractory is eliminated and should currently include lack of response to RTX and TPO-RA [115]. In 2022, Vianelli et al. defined it as the persistence of low platelet counts despite appropriate use of all conventional therapies, regardless of hemorrhagic manifestations. They reported that the threshold value to identify a “low” platelet count is variable, depending on the patient’s age, comorbidities, and concomitant therapies (e.g., antiplatelet or anticoagulant agents) [116]. In 2023, Arnold DM et al. proposed the following definition of refractory ITP for consideration and future evaluation: persistence of severe thrombocytopenia (platelets < 20 × 10^9^/L) and bleeding in a patient with ITP who has received rituximab, two different TPO-RAs, and at least one immunosuppressant medication and who does not respond or only briefly responds to high-dose corticosteroids or high-dose IVIGs (less than 7 days) [117].

Although it was traditionally considered that 20% of patients do not achieve an adequate platelet count after splenectomy or after initial and subsequent medical treatment [13], nowadays, this percentage is probably much lower and difficult to estimate due to the lack of current consensus for the definition of refractoriness. Preliminary data from the McMaster and Norwegian ITP registries demonstrate that the proportion of adult ITP patients who have received first-line therapy, rituximab, at least one TPO-RA, and splenectomy ranges from 3.1% to 4.7% [117].

The first attitude in a patient with refractory ITP is to reconsider the diagnosis, request a bone marrow study (if it has not been performed), and investigate other possible causes of thrombocytopenia. According to the opinion of some experts, when faced with an apparently refractory primary ITP, there is a 50% probability of misdiagnosis in the first place, having to considerer either a secondary ITP or another non-immune cause of thrombopenia. The management of these refractory patients is complex; they respond poorly to a variety of treatments and have greater morbidity and mortality, both hemorrhagic and infectious [115].

Many of these patients can tolerate severe thrombocytopenia (platelet counts as low as 10 × 10^9^/L) relatively well and with a near-normal quality of life, so they may prefer to live with severe thrombocytopenia rather than undergo potentially toxic treatments, thus justifying therapeutic abstention. However, in some patients with severe bleeding, high bleeding risk, or significant impairment of quality of life, the initiation of treatment should be considered in consensus with the patient [13].

In the absence of specific clinical guidelines, treatment must be individualized. Scientific evidence is limited and mostly consists of isolated cases or small series. Mycophenolate mofetil (MMF), cyclosporine (CsA), azathioprine, danazol, and dapsone may be helpful. Vinca alkaloids are not an option, as prolonged treatments are associated with neurological toxicity [13]. In these patients, instead of carrying out sequential therapy with different agents, it is more appropriate to carry out combined therapy using therapeutic strategies with non-overlapping toxicities and different mechanisms of action on the pathways involved in the pathogenesis of ITP [11]. A small retrospective study showed that in this group of patients, combined therapy with TPO-RA and immunosuppressants achieved responses in 70% of cases (7 of 10 patients) [118]. These results were corroborated in an independent study that included 18 patients with refractory primary ITP in which the combination of an immunosuppressant (CsA or MMF) and a TPO-RA (eltrombopag/romiplostim) showed an efficacy of 72% (13 of 18 patients) [119]. Another more recent study with a larger number of patients also explored this “combined therapy” with a TPO-RA (romiplostim or eltrombopag) plus immunosuppressant therapy (MMF, azathioprine, cyclophosphamide, CsA, or everolimus) in 39 adults with refractory ITP. An improvement in platelet count was observed in 77% of the cohort. This included a response in 6 patients and a complete response in 24 patients, with responses maintained for a median duration of 15 months [120]. 

## 8. New Therapies for ITP

Due to the persistence of refractory patients, research on the treatment of ITP continues to advance and is mainly directed at specific molecular targets, seeking action on the different pathophysiological mechanisms of the disease, namely decreasing the amount of circulating autoantibodies, decreasing clearance at the splenic level, decreasing hepatic platelet clearance, preventing platelet destruction by complement inhibition (fine tunning the immune response), and stimulating megakaryopoiesis (Table 3). To learn more about new molecules in the clinical development phase, the reader is referred to other more detailed reviews published recently [121,122,123,124].

### 8.1. Fine Tuning the Immune Response

Rozanolixizumab is a humanized monoclonal antibody directed specifically against the neonatal Fc receptor (FcRn). By blocking the binding of IgG to the receptor, IgG recycling is compromised, and lysosomal degradation is accelerated, subsequently reducing plasma levels of both normal and pathological IgG. In a phase II study, more than 50% of adult patients with persistent/chronic primary ITP achieved platelet responses (≥50 × 10^9^/L) on day 8 after a single subcutaneous injection of rozanolixizumab at doses of 15 and 20 mg/kg. The most common adverse event was headache, which, in most cases, was considered to be related to treatment [125]. With the success of this phase II trial, two phase III trials evaluating rozanolixizumab in persistent or chronic primary ITP were launched and began enrolling patients. However, both trials were terminated in 2022 by the company due to a strategic business decision, not a safety decision [121].

Efgartigimod is a human IgG1 antibody Fc-fragment that blocks FcRn and, by the same mechanism as the previous one, competes with IgG, favoring its elimination. In a phase III clinical trial, 22% of patients with chronic ITP receiving efgartigimod intravenously achieved sustained platelet count responses (≥50 × 10^9^/L for at least 4 of the last 6 weeks) compared with 5% of those receiving placebo [126]. Efgartigimod was well tolerated. A phase III study evaluating the drug subcutaneously is currently ongoing (NCT04687072). 

Rilzabrutinib is a new oral, reversible, and highly selective inhibitor of Bruton’s tyrosine kinase (BTK). Unlike ibrutinib, it does not affect platelet aggregation. In a phase II study in adult patients with relapsed or refractory ITP, 24 of 60 patients (40%) overall and 18 of 45 patients (40%) who had started rilzabrutinib treatment at the highest dose (400 mg twice daily) met the primary endpoint of platelet response (platelet count of at least 50 × 10^9^/L plus an increase from baseline of at least 20 × 10^9^/L). It was well tolerated with only grade 1 or 2 adverse events, mostly diarrhea and nausea [127]. A phase III clinical trial is currently ongoing (NCT04562766).

Long-lived plasma cells have also become a new therapeutic target in ITP. Two anti-CD38 molecules (daratumumab and mezagitamab) are being evaluated in phase II trials (NCT04703621 and NCT04278924).

Sutimlimab is a humanized IgG4 monoclonal antibody directed against C1s that completely inhibits the classical complement pathway. In a phase I study, 12 adult patients with chronic ITP were included, of which 42% responded (platelet count ≥ 50 × 10^9^/L), with 33% achieving a complete response. No significant treatment-related adverse events were observed [128]. A phase II study in adults with persistent/chronic ITP is currently ongoing (NCT04669600).

Oseltamivir is a drug that inhibits viral neuraminidase and, to a lesser extent, platelet neuraminidase. The inhibition of neuraminidase results in the preservation of the sialylation of platelet membrane glycoproteins, thereby preventing or blocking liver platelet destruction mediated by the interaction of desialylated platelets with the hepatic Ashwell–Morell receptor. Oseltamivir has been occasionally used in refractory ITP [129,130]. In a recent prospective phase II clinical trial, adult patients with newly diagnosed primary ITP were randomized to receive DXM (n = 47) or DXM plus oseltamivir (n = 43). The initial overall response at 14 days was significantly higher in the combination treatment group than in the DXM alone group (86% vs. 66%). The sustained response rate at 6 months in the DXM plus oseltamivir group was also significantly higher than in the DXM alone group (53% vs. 30%). There were no apparent differences between the two groups at 12 and 18 months. There was no difference in response if patients had antibodies against GPIb/IX [131].

### 8.2. Fine Tuning the Immune Response and Promoting Megakaryopoiesis

Decitabine is a demethylating agent used in the treatment of myelodysplastic syndromes that potentially reactivates silenced (i.e., methylated) genes and may aid in cell differentiation or induce cell toxicity in a dose-dependent manner. In particular, it was shown that decitabine promotes murine megakaryocyte differentiation in vitro and in vivo, as well as in vitro in human cell cultures conditioned with ITP plasma [132,133]. In a prospective study of 45 adult patients with refractory ITP, low-dose decitabine (3.5 mg/m^2^ intravenously for 3 consecutive days every 28 days (3 cycles)) showed an overall response rate of 51%, with a median time to initial response of 28 days. Sustained response rates at 6, 12, and 18 months were 44%, 31%, and 20%, respectively. Adverse events were mild, mainly nausea [134]. 

## 9. Conclusions

ITP is an autoimmune disease that causes a decrease in the number of platelets and a predisposition to bleeding. Although we currently know more about its pathophysiology, diagnosis continues to be one of exclusion. There are also no tests that allow us to distinguish the predominant pathogenic pathway involved in each patient’s disease, which results in homogeneity of the treatment strategy for all patients (trial–error). Furthermore, although we have different treatment options, we are not yet able to predict the response to each of them. Another outstanding issue is the uncertainty about which patients to treat, since the bleeding risk is variable and is not exclusively predicted by the degree of thrombocytopenia. We need better stratification of patients to avoid overtreatment, since in many cases, their side effects outweigh the complications derived from the disease. Glucocorticoids and IVIG remain the first-line treatment. Despite recent advances, most adults will require second-line therapy for which both comorbidities and the patient’s opinion must be taken into account, with TPO-RAs being the most used option currently. Although agonists have drastically changed the management of the disease, a small percentage of patients remain refractory to the usual first- and second-line therapies, which is a real concern for physicians, since therapeutic options in these cases are scarce. Thus, it is a necessity to develop not only specific therapies aimed at the different pathophysiological mechanisms of the disease but also tests that can indicate the optimal treatment options in a personalized manner. Additionally, new concepts with respect to the treatment approach for ITP patients are being considered and studied, such as combination therapies as first-line treatment, where immunosuppression and megakaryopoietic stimulation are targeted. ITP continues to present new challenges for healthcare professionals, and personalization of treatment should be the primary future goal in the management of ITP.

## Figures and Tables

**Table 1:** Historical overview of ITP treatments.

Year	Therapy
1735	Elixirium acidum halleri (citric acid)
19th century	Moderate exercise in the open air, a generous diet, and the free use of wine Purgatives
1916	First splenectomyBeginning of the splenectomy era
1951	CorticosteroidsBeginning of the immunosuppressant era
1972	First randomized double-blind study of corticosteroids versus placebo
1981	Intravenous immunoglobulins
1983	Specific anti-D immunoglobulin
1994	Dexamethasone in ITP was described for the first time
2001	First prospective study of rituximab use in chronic ITP
2008	FDA approval of romiplostim and eltrombopagBeginning of the TPO-RA era
2018	FDA approval of fostamatinib
2019	FDA approval of avatrombopag
2024	New drugs in clinical development

Abbreviations: FDA, Food and Drug Administration; TPO-RA, thrombopoietin receptor agonist. Adapted from: Stasi, R.; Newland, A. C. ITP: A Historical Perspective. British Journal of Haematology 2011, 153 (4), 437–450 [9], Imbach P, Kühne T, Signer E. Historical aspects and present knowledge of idiopathic thrombocytopenic purpura. Br J Haematol. 2002 Dec;119(4):894–900 [10].

**Table 2:** Second-line treatments for ITP.

	TPO-RA	Fostamatinib	Rituximab	Splenectomy
Recommendation	First strategy	Second strategy	Second strategy	Second strategy
Advantages	Good responses (70–95% initial, 40–60% sustained) Possibility of discontinuation Safe therapies	No risk of thrombosis or immunosuppression	Good tolerance No chronic medication No risk of thrombosis	Good responses (>80% initial, 50–75% sustained) No chronic medication Low cost
Disadvantages	Cost	Low sustained responses (18% after 24 weeks) Adverse effects (gastrointestinal effects, hypertension) Cost	Low sustained responses (20% after 5 years) Immunosuppression	Adverse effects (surgical effects, thrombosis, infections) Need for vaccination

**Table 3:** New treatments for ITP.

Description	Drug	Mechanism of Action
Recently approved treatments		
TPO-RA	Avatrombopag	Stimulates the proliferation of megakaryocytes, maturation, and production of platelets
Syk inhibitor	Fostamatinib	Decreases splenic platelet clearance
Treatments in clinical development		
FcRn inhibitors	Rozanolixizumab Efgartigimod	Decreases plasma levels of IgG (normal and pathological)
BTK inhibitor	Rilzabrutinib	Decreases splenic platelet clearance Decreases the production of autoantibodies
Anti-CD38	Daratumumab Mezagitamab	Decreases the production of autoantibodies
Complement (C1s) inhibitor	Sutimlimab	Decreases complement-dependent cytotoxicity, thereby reducing platelet destruction
Neuraminidase inhibitor	Oseltamivir	Inhibits platelet desialylation, thus decreasing hepatic platelet clearance
Epigenetic modulation	Decitabine	Promotes the maturation of megakaryocytes and platelet production Enhances Treg lymphocytes and decreases proinflammatory cytokines

Abbreviations: BTK, Bruton’s tyrosine kinase; C1s, C1 esterase; FcRn, neonatal Fc receptor; SYK: spleen tyrosine kinase; TPO-RA: thrombopoietin receptor agonists. Adapted from references [121,122,123,124].

## Data Availability

Not applicable.

## References

[1] Rodeghiero F., Stasi R., Gernsheimer T., Michel M., Provan D., Arnold D.M., Bussel J.B., Cines D.B., Chong B.H., Cooper N. (2009). Standardization of terminology, definitions and outcome criteria in immune thrombocytopenic purpura of adults and children: Report from an international working group. Blood.

[2] Terrell D.R., Beebe L.A., Vesely S.K., Neas B.R., Segal J.B., George J.N. (2010). The incidence of immune thrombocytopenic purpura in children and adults: A critical review of published reports. Am. J. Hematol..

[3] Abrahamson P.E., Hall S.A., Feudjo-Tepie M., Mitrani-Gold F.S., Logie J. (2009). The incidence of idiopathic thrombocytopenic purpura among adults: A population-based study and literature review. Eur. J. Haematol..

[4] Hedman A., Henter J.I., Hedlund I., Elinder G. (1997). Prevalence and treatment of chronic idiopathic thrombocytopenic purpura of childhood in Sweden. Acta Paediatr..

[5] Singh A., Uzun G., Bakchoul T. (2021). Primary Immune Thrombocytopenia: Novel Insights into Pathophysiology and Disease Management. J. Clin. Med..

[6] Consolini R., Legitimo A., Caparello M.C. (2016). The Centenary of Immune Thrombocytopenia—Part 1: Revising Nomenclature and Pathogenesis. Front. Pediatr..

[7] González-López T.J., Newland A., Provan D. (2023). Current Concepts in the Diagnosis and Management of Adult Primary Immune Thrombocytopenia: Our Personal View. Medicina.

[8] Martínez-Carballeira D., Bernardo Á., Caro A., Soto I., Gutiérrez L. (2024). Pathophysiology, Clinical Manifestations and Diagnosis of Immune Thrombocytopenia: Contextualization from a Historical Perspective. Hematol. Rep..

[9] Stasi R., Newland A.C. (2011). ITP: A historical perspective. Br. J. Haematol..

[10] Imbach P., Kühne T., Signer E. (2002). Historical aspects and present knowledge of idiopathic thrombocytopenic purpura. Br. J. Haematol..

[11] Mingot-Castellano M.E., Canaro Hirnyk M., Sánchez-González B., Álvarez-Román M.T., Bárez-García A., Bernardo-Gutiérrez Á., Bernat-Pablo S., Bolaños-Calderón E., Butta-Coll N., Caballero-Navarro G. (2023). Recommendations for the Clinical Approach to Immune Thrombocytopenia: Spanish ITP Working Group (GEPTI). J. Clin. Med.

[12] Neunert C., Terrell D.R., Arnold D.M., Buchanan G., Cines D.B., Cooper N., Cuker A., Despotovic J.M., George J.N., Grace R.F. (2019). American Society of Hematology 2019 guidelines for immune thrombocytopenia. Blood Adv..

[13] Provan D., Arnold D.M., Bussel J.B., Chong B.H., Cooper N., Gernsheimer T., Ghanima W., Godeau B., González-López T.J., Grainger J. (2019). Updated international consensus report on the investigation and management of primary immune thrombocytopenia. Blood Adv..

[14] Neunert C., Noroozi N., Norman G., Buchanan G.R., Goy J., Nazi I., Kelton J.G., Arnold D.M. (2015). Severe bleeding events in adults and children with primary immune thrombocytopenia: A systematic review. J. Thromb. Haemost..

[15] Cohen Y.C., Djulbegovic B., Shamai-Lubovitz O., Mozes B. (2000). The bleeding risk and natural history of idiopathic thrombocytopenic purpura in patients with persistent low platelet counts. Arch. Intern. Med..

[16] Adelborg K., Kristensen N.R., Nørgaard M., Bahmanyar S., Ghanima W., Kilpatrick K., Frederiksen H., Ekstrand C., Sørensen H.T., Fynbo Christiansen C. (2019). Cardiovascular and bleeding outcomes in a population-based cohort of patients with chronic immune thrombocytopenia. J. Thromb. Haemost..

[17] Piel-Julian M.L., Mahévas M., Germain J., Languille L., Comont T., Lapeyre-Mestre M., Payrastre B., Beyne-Rauzy O., Michel M., Godeau B. (2018). Risk factors for bleeding, including platelet count threshold, in newly diagnosed immune thrombocytopenia adults. J. Thromb. Haemost..

[18] Wintrobe M.M., Cartwright G.E., Palmer J.G., Kuhns W.J., Samuels L.T. (1951). Effect of corticotrophin and cortisone on the blood in various disorders in man. AMA Arch. Intern. Med..

[19] Andersen J.C. (1994). Response of Resistant Idiopathic Thrombocytopenic Purpura to Pulsed High-Dose Dexamethasone Therapy. N. Engl. J. Med..

[20] Zufferey A., Kapur R., Semple J. (2017). Pathogenesis and Therapeutic Mechanisms in Immune Thrombocytopenia (ITP). J. Clin. Med..

[21] Mithoowani S., Gregory-Miller K., Goy J., Miller M.C., Wang G., Noroozi N., Kelton J.G., Arnold D.M. (2016). High-dose dexamethasone compared with prednisone for previously untreated primary immune thrombocytopenia: A systematic review and meta-analysis. Lancet Haematol..

[22] Ma J., Fu L., Chen Z., Gu H., Ma J., Wu R. (2020). High-dose dexamethasone as a replacement for traditional prednisone as the first-line treatment in children with previously untreated primary immune thrombocytopenia: A prospective, randomized single-center study. Int. J. Hematol..

[23] Cooper N., Ghanima W. (2019). Immune Thrombocytopenia. N. Engl. J. Med..

[24] Wang L., Xu L., Hao H., Jansen A.J.G., Liu G., Li H., Liu X., Zhao Y., Peng J., Hou M. (2020). First line treatment of adult patients with primary immune thrombocytopenia: A real-world study. Platelets.

[25] Imbach P. (2012). Treatment of immune thrombocytopenia with intravenous immunoglobulin and insights for other diseases. Swiss. Med. Wkly..

[26] Imbach P., Barandun S., d’Apuzzo V., Baumgartner C., Hirt A., Morell A., Rossi E., Schöni M., Vest M., Wagner H.P. (1981). High-dose intravenous gammaglobulin for idiopathic thrombocytopenic purpura in childhood. Lancet.

[27] Newland A.C., Treleaven J.G., Minchinton R.M., Waters A.H. (1983). High-dose intravenous IgG in adults with autoimmune thrombocytopenia. Lancet.

[28] Newland A.C., Boots M.A., Patterson K.G. (1984). Intravenous IgG for autoimmune thrombocytopenia in pregnancy. N. Engl. J. Med..

[29] Crow A.R., Lazarus A.H. (2008). The Mechanisms of Action of Intravenous Immunoglobulin and Polyclonal Anti-D Immunoglobulin in the Amelioration of Immune Thrombocytopenic Purpura: What Do We Really Know?. Transfus. Med. Rev..

[30] Provan D., Stasi R., Newland A.C., Blanchette V.S., Bolton-Maggs P., Bussel J.B., Chong B.H., Cines D.B., Gernsheimer T.B., Godeau B. (2010). International consensus report on the investigation and management of primary immune thrombocytopenia. Blood.

[31] Godeau B., Chevret S., Varet B., Lefrère F., Zini J.M., Bassompierre F., Chèze S., Legouffe E., Hulin C., Grange M.J. (2002). Intravenous immunoglobulin or high-dose methylprednisolone, with or without oral prednisone, for adults with untreated severe autoimmune thrombocytopenic purpura: A randomised, multicentre trial. Lancet.

[32] Go R.S., Johnston K.L., Bruden K.C. (2007). The association between platelet autoantibody specificity and response to intravenous immunoglobulin G in the treatment of patients with immune thrombocytopenia. Haematologica.

[33] Peng J., Ma S.H., Liu J., Hou Y., Liu X.M., Niu T., Xu R.R., Guo C.S., Wang X.M., Cheng Y.F. (2014). Association of autoantibody specificity and response to intravenous immunoglobulin G therapy in immune thrombocytopenia: A multicenter cohort study. J. Thromb. Haemost..

[34] Li J., van der Wal D.E., Zhu G., Xu M., Yougbare I., Ma L., Vadasz B., Carrim N., Grozovsky R., Ruan M. (2015). Desialylation is a mechanism of Fc-independent platelet clearance and a therapeutic target in immune thrombocytopenia. Nat. Commun..

[35] Al-Samkari H., Rosovsky R.P., Karp Leaf R.S., Smith D.B., Goodarzi K., Fogerty A.E., Sykes D.B., Kuter D.J. (2020). A modern reassessment of glycoprotein-specific direct platelet autoantibody testing in immune thrombocytopenia. Blood Adv..

[36] Salama A., Kiefel V., Amberg R., Mueller-Eckhardt C. (1984). Treatment of autoimmune thrombocytopenic purpura with rhesus antibodies (anti-Rh0(D). Blut.

[37] Salama A., Mueller-Eckhardt C., Kiefel V. (1983). Effect of intravenous immunoglobulin in immune thrombocytopenia. Lancet.

[38] Bussel J.B., Graziano J.N., Kimberly R.P., Pahwa S., Aledort L.M. (1991). Intravenous anti-D treatment of immune thrombocytopenic purpura: Analysis of efficacy, toxicity, and mechanism of effect. Blood.

[39] Cooper N., Woloski B.M.R., Fodero E.M., Novoa M., Leber M., Beer J.H., Bussel J.B. (2002). Does treatment with intermittent infusions of intravenous anti-D allow a proportion of adults with recently diagnosed immune thrombocytopenic purpura to avoid splenectomy?. Blood.

[40] Kane I., Ragucci D., Shatat I.F., Bussel J., Kalpatthi R. (2010). Comparison of intravenous immune globulin and high dose anti-D immune globulin as initial therapy for childhood immune thrombocytopenic purpura. Br. J. Haematol..

[41] Michel M., Novoa M.V., Bussel J.B. (2003). Intravenous anti-D as a treatment for immune thrombocytopenic purpura (ITP) during pregnancy. Br. J. Haematol..

[42] Newman G.C., Novoa M.V., Fodero E.M., Lesser M.L., Woloski B.M., Bussel J.B. (2001). A dose of 75 microg/kg/d of i.v. anti-D increases the platelet count more rapidly and for a longer period of time than 50 microg/kg/d in adults with immune thrombocytopenic purpura. Br. J. Haematol..

[43] Scaradavou A., Woo B., Woloski B.M., Cunningham-Rundles S., Ettinger L.J., Aledort L.M., Bussel J.B. (1997). Intravenous anti-D treatment of immune thrombocytopenic purpura: Experience in 272 patients. Blood.

[44] Lazarus A.H., Crow A.R. (2003). Mechanism of action of IVIG and anti-D in ITP. Transfus. Apher. Sci..

[45] Naithani R., Kumar R., Mahapatra M., Tyagi S., Saxena R. (2009). Efficacy and safety of anti-D for treatment of adults with immune thrombocytopenia. Platelets.

[46] Cheung E., Liebman H.A. (2009). Anti-RhD immunoglobulin in the treatment of immune thrombocytopenia. Biologics.

[47] Gaines A.R. (2005). Disseminated intravascular coagulation associated with acute hemoglobinemia or hemoglobinuria following Rh(0)(D) immune globulin intravenous administration for immune thrombocytopenic purpura. Blood.

[48] Tarantino M.D., Bussel J.B., Cines D.B., McCrae K.R., Gernsheimer T., Liebman H.A., Wong W.-Y., Kulkarni R., Grabowski E., McMillan R. (2007). A closer look at intravascular hemolysis (IVH) following intravenous anti-D for immune thrombocytopenic purpura (ITP). Blood.

[49] Kaushansky K. (2015). Thrombopoiesis. Semin. Hematol..

[50] Bartley T.D., Bogenberger J., Hunt P., Li Y.S., Lu H.S., Martin F., Chang M.S., Samal B., Nichol J.L., Swift S. (1994). Identification and cloning of a megakaryocyte growth and development factor that is a ligand for the cytokine receptor Mpl. Cell.

[51] Kuter D.J., Beeler D.L., Rosenberg R.D. (1994). The purification of megapoietin: A physiological regulator of megakaryocyte growth and platelet production. Proc. Natl. Acad. Sci. USA.

[52] Lok S., Kaushansky K., Holly R.D., Kuijper J.L., Lofton-Day C.E., Oort P.J., Grant F.J., Heipel M.D., Burkhead S.K., Kramer J.M. (1994). Cloning and expression of murine thrombopoietin cDNA and stimulation of platelet production in vivo. Nature.

[53] Kuter D.J. (2013). The biology of thrombopoietin and thrombopoietin receptor agonists. Int. J. Hematol..

[54] Emmons R.V., Reid D.M., Cohen R.L., Meng G., Young N.S., Dunbar C.E., Shulman N.R. (1996). Human thrombopoietin levels are high when thrombocytopenia is due to megakaryocyte deficiency and low when due to increased platelet destruction. Blood.

[55] Kosugi S., Kurata Y., Tomiyama Y., Tahara T., Kato T., Tadokoro S., Shiraga M., Honda S., Kanakura Y., Matsuzawa Y. (1996). Circulating thrombopoietin level in chronic immune thrombocytopenic purpura. Br. J. Haematol..

[56] Ghanima W., Cooper N., Rodeghiero F., Godeau B., Bussel J.B. (2019). Thrombopoietin receptor agonists: Ten years later. Haematologica.

[57] Nplate Epar. https://www.ema.europa.eu/en/documents/product-information/nplate-epar-product-information_en.pdf.

[58] Revolade Epar. https://www.ema.europa.eu/en/documents/product-information/revolade-epar-product-information_en.pdf.

[59] Rodeghiero F., Carli G. (2017). Beyond immune thrombocytopenia: The evolving role of thrombopoietin receptor agonists. Ann. Hematol..

[60] Kuter D.J., Bussel J.B., Lyons R.M., Pullarkat V., Gernsheimer T.B., Senecal F.M., Aledort L.M., George J.N., Kessler C.M., Sanz M.A. (2008). Efficacy of romiplostim in patients with chronic immune thrombocytopenic purpura: A double-blind randomised controlled trial. Lancet.

[61] Kuter D.J., Bussel J.B., Newland A., Baker R.I., Lyons R.M., Wasser J., Viallard J.-F., Macik G., Rummel M., Nie K. (2013). Long-term treatment with romiplostim in patients with chronic immune thrombocytopenia: Safety and efficacy. Br. J. Haematol..

[62] Cines D.B., Wasser J., Rodeghiero F., Chong B.H., Steurer M., Provan D., Lyons R., Garcia-Chavez J., Carpenter N., Wang X. (2017). Safety and efficacy of romiplostim in splenectomized and nonsplenectomized patients with primary immune thrombocytopenia. Haematologica.

[63] Bussel J.B., Provan D., Shamsi T., Cheng G., Psaila B., Kovaleva L., Salama A., Jenkins J.M., Roychowdhury D., Mayer B. (2009). Effect of eltrombopag on platelet counts and bleeding during treatment of chronic idiopathic thrombocytopenic purpura: A randomised, double-blind, placebo-controlled trial. Lancet.

[64] Cheng G., Saleh M.N., Marcher C., Vasey S., Mayer B., Aivado M., Arning M., Stone N.L., Bussel J.B. (2011). Eltrombopag for management of chronic immune thrombocytopenia (RAISE): A 6-month, randomised, phase 3 study. Lancet.

[65] Wong R.S.M., Saleh M.N., Khelif A., Salama A., Portella M.S.O., Burgess P., Bussel J.B. (2017). Safety and efficacy of long-term treatment of chronic/persistent ITP with eltrombopag: Final results of the EXTEND study. Blood.

[66] Bussel J.B., Buchanan G.R., Nugent D.J., Gnarra D.J., Bomgaars L.R., Blanchette V.S., Wang Y.-M., Nie K., Jun S. (2011). A randomized, double-blind study of romiplostim to determine its safety and efficacy in children with immune thrombocytopenia. Blood.

[67] Elalfy M.S., Abdelmaksoud A.A., Eltonbary K.Y. (2011). Romiplostim in children with chronic refractory ITP: Randomized placebo controlled study. Ann. Hematol..

[68] Tarantino M.D., Bussel J.B., Blanchette V.S., Beam D., Roy J., Despotovic J., Raj A., Carpenter N., Mehta B., Eisen M. (2019). Long-term treatment with romiplostim and treatment-free platelet responses in children with chronic immune thrombocytopenia. Haematologica.

[69] Tarantino M.D., Bussel J.B., Blanchette V.S., Despotovic J., Bennett C., Raj A., Williams B., Beam D., Morales J., Rose M.J. (2016). Romiplostim in children with immune thrombocytopenia: A phase 3, randomised, double-blind, placebo-controlled study. Lancet.

[70] Bussel J.B., de Miguel P.G., Despotovic J.M., Grainger J.D., Sevilla J., Blanchette V.S., Krishnamurti L., Connor P., David M., Boayue K.B. (2015). Eltrombopag for the treatment of children with persistent and chronic immune thrombocytopenia (PETIT): A randomised, multicentre, placebo-controlled study. Lancet Haematol..

[71] Grainger J.D., Locatelli F., Chotsampancharoen T., Donyush E., Pongtanakul B., Komvilaisak P., Sosothikul D., Drelichman G., Sirachainan N., Holzhauer S. (2015). Eltrombopag for children with chronic immune thrombocytopenia (PETIT2): A randomised, multicentre, placebo-controlled trial. Lancet.

[72] Rodeghiero F., Stasi R., Giagounidis A., Viallard J.-F., Godeau B., Pabinger I., Cines D., Liebman H., Wang X., Woodard P. (2013). Long-term safety and tolerability of romiplostim in patients with primary immune thrombocytopenia: A pooled analysis of 13 clinical trials. Eur. J. Haematol..

[73] González-Porras J.R., Mingot-Castellano M.E., Andrade M.M., Alonso R., Caparrós I., Arratibel M.C., Fernández-Fuertes F., Cortti M.J., Pascual C., Sánchez-González B. (2015). Use of eltrombopag after romiplostim in primary immune thrombocytopenia. Br. J. Haematol..

[74] Khellaf M., Viallard J.-F., Hamidou M., Cheze S., Roudot-Thoraval F., Lefrere F., Fain O., Audia S., Abgrall J.-F., Michot J.-M. (2013). A retrospective pilot evaluation of switching thrombopoietic receptor-agonists in immune thrombocytopenia. Haematologica.

[75] Kuter D.J., Macahilig C., Grotzinger K.M., Poston S.A., Wang P.F., Dawson K.L., Ward M. (2015). Treatment patterns and clinical outcomes in patients with chronic immune thrombocytopenia (ITP) switched to eltrombopag or romiplostim. Int. J. Hematol..

[76] Cantoni S., Carpenedo M., Mazzucconi M.G., De Stefano V., Carrai V., Ruggeri M., Specchia G., Vianelli N., Pane F., Consoli U. (2018). Alternate use of thrombopoietin receptor agonists in adult primary immune thrombocytopenia patients: A retrospective collaborative survey from Italian hematology centers. Am. J. Hematol..

[77] Lozano M.L., Mingot-Castellano M.E., Perera M.M., Jarque I., Campos-Alvarez R.M., González-López T.J., Carreño-Tarragona G., Bermejo N., Lopez-Fernandez M.F., de Andrés A. (2019). Deciphering predictive factors for choice of thrombopoietin receptor agonist, treatment free responses, and thrombotic events in immune thrombocytopenia. Sci. Rep..

[78] González-López T.J., Pascual C., Álvarez-Román M.T., Fernández-Fuertes F., Sánchez-González B., Caparrós I., Jarque I., Mingot-Castellano M.E., Hernández-Rivas J.A., Martín-Salces M. (2015). Successful discontinuation of eltrombopag after complete remission in patients with primary immune thrombocytopenia. Am. J. Hematol..

[79] Mahévas M., Fain O., Ebbo M., Roudot-Thoraval F., Limal N., Khellaf M., Schleinitz N., Bierling P., Languille L., Godeau B. (2014). The temporary use of thrombopoietin-receptor agonists may induce a prolonged remission in adult chronic immune thrombocytopenia. Results of a French observational study. Br. J. Haematol..

[80] Jurczak W., Chojnowski K., Mayer J., Krawczyk K., Jamieson B.D., Tian W., Allen L.F. (2018). Phase 3 randomised study of avatrombopag, a novel thrombopoietin receptor agonist for the treatment of chronic immune thrombocytopenia. Br. J. Haematol..

[81] Doptelet Epar. https://www.ema.europa.eu/en/documents/product-information/doptelet-epar-product-information_en.pdf.

[82] Al-Samkari H., Jiang D., Gernsheimer T., Liebman H., Lee S., Wojdyla M., Vredenburg M., Cuker A. (2022). Adults with immune thrombocytopenia who switched to avatrombopag following prior treatment with eltrombopag or romiplostim: A multicentre US study. Br. J. Haematol..

[83] Bussel J., Arnold D.M., Grossbard E., Mayer J., Treliński J., Homenda W., Hellmann A., Windyga J., Sivcheva L., Khalafallah A.A. (2018). Fostamatinib for the treatment of adult persistent and chronic immune thrombocytopenia: Results of two phase 3, randomized, placebo-controlled trials. Am. J. Hematol..

[84] Boccia R., Cooper N., Ghanima W., Boxer M.A., Hill Q.A., Sholzberg M., Tarantino M.D., Todd L.K., Tong S., Bussel J.B. (2020). Fostamatinib is an effective second-line therapy in patients with immune thrombocytopenia. Br. J. Haematol..

[85] Tavlesse Epar. https://www.ema.europa.eu/en/documents/product-information/tavlesse-epar-product-information_en.pdf.

[86] Cooper N., Ghanima W., Hill Q.A., Nicolson P.L., Markovtsov V., Kessler C. (2023). Recent advances in understanding spleen tyrosine kinase (SYK) in human biology and disease, with a focus on fostamatinib. Platelets.

[87] Martinez-Botia P., Meinders M., De Cuyper I.M., Eble J.A., Semple J.W., Gutierrez L. (2022). Dissecting platelet proteomics to understand the pathophysiology of immune thrombocytopenia: Studies in mouse models. Blood Adv..

[88] Lucchesi A., Fattizzo B., De Stefano V., Ruggeri M., Siragusa S., Vianelli N., Zaja F., Rodeghiero F. (2023). Use and positioning of fostamatinib in the management of primary chronic immune thrombocytopenia: An Italian expert opinion. Ther. Adv. Hematol..

[89] Mabthera Epar. https://www.ema.europa.eu/en/documents/product-information/mabthera-epar-product-information_en.pdf.

[90] Lucchini E., Zaja F., Bussel J. (2019). Rituximab in the treatment of immune thrombocytopenia: What is the role of this agent in 2019?. Haematologica.

[91] Press O.W., Appelbaum F., Ledbetter J.A., Martin P.J., Zarling J., Kidd P., Thomas E.D. (1987). Monoclonal antibody 1F5 (anti-CD20) serotherapy of human B cell lymphomas. Blood.

[92] Lee E.J., Kueck B. (1998). Rituxan in the treatment of cold agglutinin disease. Blood.

[93] Shvidel L., Klepfish A., Berrebi A. (2001). Successful treatment with Rituximab for relapsing immune thrombocytopenic purpura (ITP) associated with low-grade non-Hodgkin’s lymphoma. Am. J. Hematol..

[94] Stasi R., Pagano A., Stipa E., Amadori S. (2001). Rituximab chimeric anti-CD20 monoclonal antibody treatment for adults with chronic idiopathic thrombocytopenic purpura. Blood.

[95] Wang J., Wiley J.M., Luddy R., Greenberg J., Feuerstein M.A., Bussel J.B. (2005). Chronic immune thrombocytopenic purpura in children: Assessment of rituximab treatment. J. Pediatr..

[96] Patel V.L., Mahévas M., Lee S.Y., Stasi R., Cunningham-Rundles S., Godeau B., Kanter J., Neufeld E., Taube T., Ramenghi U. (2012). Outcomes 5 years after response to rituximab therapy in children and adults with immune thrombocytopenia. Blood.

[97] Dong Y., Yue M., Hu M. (2021). The Efficacy and Safety of Different Dosages of Rituximab for Adults with Immune Thrombocytopenia: A Systematic Review and Meta-Analysis. BioMed Res. Int..

[98] Li Y., Shi Y., He Z., Chen Q., Liu Z., Yu L., Wang C. (2019). The efficacy and safety of low-dose rituximab in immune thrombocytopenia: A systematic review and meta-analysis. Platelets.

[99] Khellaf M., Charles-Nelson A., Fain O., Terriou L., Viallard J.-F., Cheze S., Graveleau J., Slama B., Audia S., Ebbo M. (2014). Safety and efficacy of rituximab in adult immune thrombocytopenia: Results from a prospective registry including 248 patients. Blood.

[100] Mahévas M., Ebbo M., Audia S., Bonnotte B., Schleinitz N., Durand J.-M., Chiche L., Khellaf M., Bierling P., Roudot-Thoraval F. (2013). Efficacy and safety of rituximab given at 1,000 mg on days 1 and 15 compared to the standard regimen to treat adult immune thrombocytopenia. Am. J. Hematol..

[101] Zaja F., Baccarani M., Mazza P., Bocchia M., Gugliotta L., Zaccaria A., Vianelli N., Defina M., Tieghi A., Amadori S. (2010). Dexamethasone plus rituximab yields higher sustained response rates than dexamethasone monotherapy in adults with primary immune thrombocytopenia. Blood.

[102] Gudbrandsdottir S., Birgens H.S., Frederiksen H., Jensen B.A., Jensen M.K., Kjeldsen L., Klausen T.W., Larsen H., Mourits-Andersen H.T., Nielsen C.H. (2013). Rituximab and dexamethasone vs. dexamethasone monotherapy in newly diagnosed patients with primary immune thrombocytopenia. Blood.

[103] Bussel J.B., Lee C.S., Seery C., Imahiyerobo A.A., Thompson M.V., Catellier D., Turenne I.G., Patel V.L., Basciano P.A., Elstrom R.L. (2014). Rituximab and three dexamethasone cycles provide responses similar to splenectomy in women and those with immune thrombocytopenia of less than two years duration. Haematologica.

[104] Chugh S., Darvish-Kazem S., Lim W., Crowther M.A., Ghanima W., Wang G., Heddle N.M., Kelton J.G., Arnold D.M. (2015). Rituximab plus standard of care for treatment of primary immune thrombocytopenia: A systematic review and meta-analysis. Lancet Haematol..

[105] Kaznelson P. (1916). Verschwinden der hamorrhagischen diathese bei einem falle von essentieller thrombopenie (frank) nach milzexstiparation: Splenogene thrombolytische purpura. Wien. Klin. Wochenschr..

[106] Harrington W.J., Minnich V., Hollingsworth J.W., Moore C.V. (1951). Demonstration of a thrombocytopenic factor in the blood of patients with thrombocytopenic purpura. J. Lab. Clin. Med..

[107] Palandri F., Polverelli N., Sollazzo D., Romano M., Catani L., Cavo M., Vianelli N. (2016). Have splenectomy rate and main outcomes of ITP changed after the introduction of new treatments? A monocentric study in the outpatient setting during 35 years. Am. J. Hematol..

[108] Chaturvedi S., Arnold D.M., McCrae K.R. (2018). Splenectomy for immune thrombocytopenia: Down but not out. Blood.

[109] Kojouri K., Vesely S.K., Terrell D.R., George J.N. (2004). Splenectomy for adult patients with idiopathic thrombocytopenic purpura: A systematic review to assess long-term platelet count responses, prediction of response, and surgical complications. Blood.

[110] Boyle S., White R.H., Brunson A., Wun T. (2013). Splenectomy and the incidence of venous thromboembolism and sepsis in patients with immune thrombocytopenia. Blood.

[111] Gonzalez-Porras J.R., Escalante F., Pardal E., Sierra M., Garcia-Frade L.J., Redondo S., Arefi M., Aguilar C., Ortega F., De Cabo E. (2013). Safety and efficacy of splenectomy in over 65-yrs-old patients with immune thrombocytopenia. Eur. J. Haematol..

[112] Mageau A., Terriou L., Ebbo M., Souchaud-Debouverie O., Orvain C., Graveleau J., Lega J.-C., Ruivard M., Viallard J.-F., Cheze S. (2022). Splenectomy for primary immune thrombocytopenia revisited in the era of thrombopoietin receptor agonists: New insights for an old treatment. Am. J. Hematol..

[113] Moulis G., Germain J., Comont T., Brun N., Dingremont C., Castel B., Arista S., Sailler L., Lapeyre-Mestre M., Beyne-Rauzy O. (2017). Newly diagnosed immune thrombocytopenia adults: Clinical epidemiology, exposure to treatments, and evolution. Results of the CARMEN multicenter prospective cohort. Am. J. Hematol..

[114] Imbach P., Kühne T., Müller D., Berchtold W., Zimmerman S., Elalfy M., Buchanan G.R. (2006). Childhood ITP: 12 months follow-up data from the prospective registry I of the Intercontinental Childhood ITP Study Group (ICIS). Pediatr. Blood Cancer.

[115] Miltiadous O., Hou M., Bussel J.B. (2020). Identifying and treating refractory ITP: Difficulty in diagnosis and role of combination treatment. Blood.

[116] Vianelli N., Auteri G., Buccisano F., Carrai V., Baldacci E., Clissa C., Bartoletti D., Giuffrida G., Magro D., Rivolti E. (2022). Refractory primary immune thrombocytopenia (ITP): Current clinical challenges and therapeutic perspectives. Ann. Hematol..

[117] Arnold D.M., Clerici B., Ilicheva E., Ghanima W. (2023). Refractory immune thrombocytopenia in adults: Towards a new definition. Br. J. Haematol..

[118] Mahévas M., Gerfaud-Valentin M., Moulis G., Terriou L., Audia S., Guenin S., Le Guenno G., Salles G., Lambotte O., Limal N. (2016). Characteristics, outcome, and response to therapy of multirefractory chronic immune thrombocytopenia. Blood.

[119] Gudbrandsdottir S., Leven E., Imahiyerobo A., Lee C.S., Bussel J. (2020). Combination of thrombopoietin receptor agonists, immunosuppressants and intravenous immunoglobulin as treatment of severe refractory immune thrombocytopenia in adults and children. Br. J. Haematol..

[120] Crickx E., Ebbo M., Rivière E., Souchaud-Debouverie O., Terriou L., Audia S., Ruivard M., Asli B., Marolleau J.-P., Méaux-Ruault N. (2023). Combining thrombopoietin receptor agonists with immunosuppressive drugs in adult patients with multirefractory immune thrombocytopenia, an update on the French experience. Br. J. Haematol..

[121] Al-Samkari H., Neufeld E.J. (2023). Novel therapeutics and future directions for refractory immune thrombocytopenia. Br. J. Haematol..

[122] Audia S., Bonnotte B. (2021). Emerging Therapies in Immune Thrombocytopenia. J. Clin. Med..

[123] Kuter D.J. (2022). Novel therapies for immune thrombocytopenia. Br. J. Haematol..

[124] Provan D., Semple J.W. (2022). Recent advances in the mechanisms and treatment of immune thrombocytopenia. EBioMedicine.

[125] Robak T., Kazmierczak M., Jarque I., Musteata V., Trelinski J., Cooper N., Kiessling P., Massow U., Woltering F., Snipes R. (2020). Phase 2 multiple-dose study of an FcRn inhibitor, rozanolixizumab, in patients with primary immune thrombocytopenia. Blood Adv..

[126] Broome C.M., McDonald V., Miyakawa Y., Carpenedo M., Kuter D.J., Al-Samkari H., Bussel J.B., Godar M., Ayguasanosa J., De Beuf K. (2023). Efficacy and safety of the neonatal Fc receptor inhibitor efgartigimod in adults with primary immune thrombocytopenia (ADVANCE IV): A multicentre, randomised, placebo-controlled, phase 3 trial. Lancet.

[127] Kuter D.J., Efraim M., Mayer J., Trneny M., McDonald V., Bird R., Regenbogen T., Garg M., Kaplan Z., Tzvetkov N. (2022). Rilzabrutinib, an Oral BTK Inhibitor, in Immune Thrombocytopenia. N. Engl. J. Med..

[128] Broome C.M., Roth A., Kuter D.J., Scully M., Smith R., Wang J., Reuter C., Hobbs W., Daak A. (2023). Safety and efficacy of classical complement pathway inhibition with sutimlimab in chronic immune thrombocytopenia. Blood Adv..

[129] Alvarez-Roman M.T., Rivas Pollmar M.I., Bernardino J.I., Lozano M.L., Martin-Salces M., Fernandez-Bello I., Jimenez-Yuste V., Butta N.V. (2016). Thrombopoietin receptor agonists in conjunction with oseltamivir for immune thrombocytopenia. AIDS.

[130] Shao L., Wu Y., Zhou H., Qin P., Ni H., Peng J., Hou M. (2015). Successful treatment with oseltamivir phosphate in a patient with chronic immune thrombocytopenia positive for anti-GPIb/IX autoantibody. Platelets.

[131] Sun L., Wang J., Shao L., Yuan C., Zhao H., Li D., Wang Z., Han P., Yu Y., Xu M. (2021). Dexamethasone plus oseltamivir versus dexamethasone in treatment-naive primary immune thrombocytopenia: A multicentre, randomised, open-label, phase 2 trial. Lancet Haematol..

[132] Wang J., Yi Z., Wang S., Li Z. (2011). The effect of decitabine on megakaryocyte maturation and platelet release. Thromb. Haemost..

[133] Zhou H., Hou Y., Liu X., Qiu J., Feng Q., Wang Y., Zhang X., Min Y., Shao L., Liu X. (2015). Low-dose decitabine promotes megakaryocyte maturation and platelet production in healthy controls and immune thrombocytopenia. Thromb. Haemost..

[134] Zhou H., Qin P., Liu Q., Yuan C., Hao Y., Zhang H., Wang Z., Ran X., Chu X., Yu W. (2019). A prospective, multicenter study of low dose decitabine in adult patients with refractory immune thrombocytopenia. Am. J. Hematol..

